# Editorial: Microenvironment-Derived Stem Cell Plasticity

**DOI:** 10.3389/fcell.2017.00082

**Published:** 2017-09-21

**Authors:** Jelena Krstic, Marietta Herrmann, Ivana Gadjanski, Slavko Mojsilovic

**Affiliations:** ^1^Institute of Cell Biology, Histology and Embryology, Medical University Graz Graz, Austria; ^2^Institute for Medical Research, University of Belgrade Belgrade, Serbia; ^3^AO Research Institute Davos, Switzerland; ^4^IZKF Group Tissue Regeneration in Musculoskeletal Diseases, Orthopedic Center for Musculoskeletal Research, University of Würzburg Würzburg, Germany; ^5^Belgrade Metropolitan University Belgrade, Serbia; ^6^Biosense Institute, University of Novi Sad Novi Sad, Serbia

**Keywords:** plasticity, stem cells, microenvironment, imaging, extracellular vesicles (EVs), oxygen tension, tissue regeneration, immunomodulation

Plasticity is the hallmark of stem cells. At the same time, stem cells, like any other cell type, are influenced by their microenvironment and respond to it accordingly. A specific microenvironment is defined by a variety of factors, including biological and chemical factors, cell-cell interactions, but also metabolic and mechanical cues. Such dynamic and specialized microenvironment where the stem cells reside is considered a stem cell niche. Tissue injury as well as malignant tissue alterations lead to changes in the niche influencing the plasticity and biology of residing stem cells. Similarly, the niche changes upon tissue damage, which eventually induces differentiation of stem cells and ultimately regeneration of the tissue.

Within this research topic, different aspects of microenvironment-derived stem cell plasticity are discussed including techniques to detect niche-residing stem cells, cell communication within the niche and the influence of oxygen tension and inflammation on stem cell plasticity. Furthermore, disease-associated microenvironments are addressed. Finally, two contributions focus on the use of micro environmental cues for therapeutic application of stem cells.

Interactions of hematopoietic stem/progenitor cells with their microenvironment is central for regulation of hematopoiesis, both in steady state and in disease. Development of imaging techniques brings about powerful new methodologies to enlighten these very complex and dynamic processes. Morikawa and Takubo concisely summarize methods used for visualization, tracking and localization of hematopoietic stem cells within their microenvironment, and review recent advances in our understanding of stem cell/niche biology through examples of imaging-based studies of various physiological and pathological hematopoietic activities.

An overview of the communication between mesenchymal stem cells (MSCs) and other cells in their microenvironment via extracellular vesicles (EVs) is provided by Dostert et al. The two-way interaction has been considered, describing MSCs as donors and recipients of EVs. A number of factors discovered in EVs have been distinguished as protein effectors and micro RNAs involved in cell-cell communication, specifically focusing on EV-mediated tissue regeneration, immunomodulation and cross talk with cancer cells.

Oxygen tension is an important aspect of the tissue microenvironment. Miclau et al. have summarized the current literature on how oxygen directs MSC fate in the context of fracture healing. There is clear evidence from preclinical and clinical studies, that a disrupted vasculature and/or ischemic conditions due to co-morbidities have a negative impact on fracture healing. However, on a cellular level, it is yet not clear how the tissue oxygen level is associated with stem cell differentiation; unexpected results have also been reported in the context of tissue revascularization indicating the need to better understand the regulation of oxygenation in fracture healing.

It has been shown that MSCs have immunomodulatory properties and the ability to adapt to the tissue environment. Tumor necrosis factor α (TNF-α) and transforming growth factor β (TGF-β) are two cytokines present in different niches, which may have opposing roles (pro- and anti-inflammatory). Lerrer et al. demonstrate in their study that these cytokines cooperatively induce a pro-inflammatory fate in MSCs. This implies that the complex composition of the niche-specific secretome is of critical importance for the immunomodulatory fate of stem cells. Michael et al. provide in their mini review very interesting aspects on the specific outcomes of inflammatory incidents affected by direct regulation of stem cells. During tissue damage caused by either pathogen infection or physical/chemical damage (sterile induced inflammation), the inflammatory response acts as a regulator of tissue stemness either by directly affecting tissue stem cells or by shifting differentiated cells toward a stem-like cell character. Menendez and Alarcón present a threshold model for aging and cancer, based on regulation of cellular reprogramming by senescence-inflammatory signaling, and offer new therapeutic strategies for these ailments. According to this model, a transient cellular reprogramming induced by activation of innate immunity or senescence-associated inflammatory components brings cells to a state of epigenetic plasticity, enabling adaptive phenotypic adjustments in order to promote damage repair and tissue regeneration. However, if this de-differentiation is not accompanied by re-acquisition of the original or alternative differentiated cell fate, the resulting tissue plasticity impairs the replacement of damaged cells and leads to chronically unresolved tissue damage.

Malignant tissue alterations such as cancer are associated with drastic changes in the tissue environment and its metabolome. In this context, the metabolic plasticity of cancer cells has been reported by Johnson et al. who analyzed the expression of the monocarboxylate transporter 1 (MCT1) in a significant number of breast cancer tissue samples with variable characteristics in terms of hormone-dependence. They showed that MCT1 level of expression varies between cancer subtypes and that it can be correlated to breast cancer recurrence, thus suggesting MCT1 as a prognostic biomarker as well as a target worth exploring for cancer therapy.

Stem cell plasticity can also be used as a tool to improve the regenerative capacity of cells. Such approach is presented by Ghosh et al., who investigated how TGF-β pretreatment affects MSC-mediated wound healing. In the mouse model, TGF-β-pretreated MSCs distribute more evenly in the wound and improve wound closure. Based on *in vitro* findings, the authors propose a time-dependent model of TGF-β-stimulated bone marrow MSCs that helps the wound healing process. They suggest that increased MSC adhesion and migration properties are regulated via integrin/focal adhesion kinase activation which is essential for both SMAD3-dependent and -independent signal transduction after TGF-β treatment.

Finally, Ottoboni et al. review the literature on neural stem cell (NSC) plasticity and methods for manipulation of their plasticity for treatment of central nervous system (CNS) diseases. They particularly emphasize the double-side effect that plasticity can have, even threatening the NSC therapeutic capacity, because manipulation of NSCs or of their trophic microenvironment might induce unwanted side effects, such as senescence. The authors conclude that it is necessary to increase the knowledge on (i) the influence of cell metabolism on NSC behavior during CNS diseases; (ii) the *ex-vivo* maintenance of NSCs; (iii) the most suitable window time for transplant to best leverage graft survival and disease specific environmental conditions. While this review was focused on neural stem cells, the mentioned aspects similarly present a challenge in other research fields.

## Author contributions

All authors JK, MH, IG, and SM contributed equally in writing the Editorial.

### Conflict of interest statement

The authors declare that the research was conducted in the absence of any commercial or financial relationships that could be construed as a potential conflict of interest.

